# 28835 Describing Physical Symptoms among Patients with PTSD at an Anxiety Clinic in Puerto Rico

**DOI:** 10.1017/cts.2021.697

**Published:** 2021-03-30

**Authors:** Marie Torres-Valentin, Karen G. Martinez-Gonzalez, Alfonso Martinez-Taboas

**Affiliations:** 1 University of Puerto Rico, Medical Sciences Campus; 2 Interamerican University of Puerto Rico, Metropolitan Campus

## Abstract

ABSTRACT IMPACT: Our work will provide valuable information about the associations between physical symptoms and PTSD in patients from a Spanish-speaking, evidence-based clinic. OBJECTIVES/GOALS: In this reserach study, we want to describe physical symptoms of patients with a preliminary PTSD diagnosis. We also want to explain associations between physical symptoms, and the presence, or absence of PTSD, and to evaluate findings in terms of prevention services, referrals, and alternatives for augmenting treatment-adherence. METHODS/STUDY POPULATION: This was a descriptive, secondary database analysis of the Center for the Study of Fear and Anxiety (by its Spanish acronym, CETMA). The database included information of the initial evaluation between 2012 and 2019. We aimed to describe sociodemographic and medical variables, and evaluate associations, in terms of the presence or absence of PTSD. RESULTS/ANTICIPATED RESULTS: Patients with PTSD were mostly women, single, with a completed bachelor’s degree. The majority had at least one neurological, or musculoskeletal condition. Respiratory conditions were the least represented. We found significant associations between musculoskeletal, neurological, and ear/nose/throat conditions, in terms of PTSD diagnosis. DISCUSSION/SIGNIFICANCE OF FINDINGS: Puerto Rico recently experienced two hurricanes, several earthquakes, and the pandemic. Findings provide data about the interface between mental and physical symptoms of patients with PTSD. We recommend a randomized population study with mental and physical variables, for understanding possible effects of cumulative stress in Puerto Ricans.

